# Analysis of Transcriptome Differences Between Subcutaneous and Intramuscular Adipose Tissue of Tibetan Pigs

**DOI:** 10.3390/genes16030246

**Published:** 2025-02-20

**Authors:** Xinming Li, Qiuyan Huang, Fanming Meng, Chun Hong, Baohong Li, Yecheng Yang, Zixiao Qu, Junda Wu, Fei Li, Haiyun Xin, Bin Hu, Jie Wu, Chuanhuo Hu, Xiangxing Zhu, Dongsheng Tang, Zongliang Du, Sutian Wang

**Affiliations:** 1 State Key Laboratory of Swine and Poultry Breeding Industry, Guangdong Provincial Key Laboratory of Animal Breeding and Nutrition, Institute of Animal Science, Guangdong Academy of Agricultural Sciences, Guangzhou 510640, Chinajiewu_email@163.com (J.W.); dzliang1008@sina.com (Z.D.); 2 Guangxi Key Laboratory of Animal Breeding, Disease Control and Prevention, College of Animal Science and Technology, Guangxi University, Nanning 530004, China; 3 School of Medicine, Foshan University, Foshan 528000, China

**Keywords:** Tibetan pig, fat deposition, subcutaneous fat, intramuscular fat, RNA-seq

## Abstract

**Background/Objectives**: Fat deposition traits in pigs directly influence pork flavor, tenderness, and juiciness and are closely linked to overall pork quality. The Tibetan pig, an indigenous breed in China, not only possesses a high intramuscular fat content but also exhibits a unique fat metabolism pattern due to long-term adaptation to harsh environments. This makes it an excellent genetic and physiological model for investigating fat deposition characteristics. Adipose tissue from different body regions displays varying morphologies, cytokines, and adipokines. This study aimed to examine adipose tissue deposition characteristics in different parts of Tibetan pigs and provide additional data to explore the underlying mechanisms of differential fat deposition. **Methods**: Our research identified significant differences in the morphology and gene expression patterns between subcutaneous fat (abdominal fat [AF] and back fat [BF]) and intramuscular fat (IMF) in Tibetan pigs. **Results**: Histological observations revealed that subcutaneous fat cells were significantly larger in area and diameter compared to IMF cells. The transcriptomic analysis further identified differentially expressed genes (DEGs) between subcutaneous fat and IMF, with a total of 65 DEGs in BF vs. IMF and 347 DEGs in AF vs. IMF, including 25 DEGs common to both comparisons. Gene Ontology (GO) and Kyoto Encyclopedia of Genes and Genomes (KEGG) enrichment analyses indicated that these genes were significantly associated with lipid metabolism-related signaling pathways, such as the Wnt, mTOR, and PI3K-Akt signaling pathways. Several DEGs, including *DDAH1*, *ADRA1B*, *SLCO3A1*, and *THBS3*, may be linked to the differences in fat deposition in different parts of Tibetan pigs, thereby affecting meat quality and nutritional value. **Conclusions**: These findings provide new insights into the unique fat distribution and deposition characteristics of Tibetan pigs and establish a foundation for breeding strategies aimed at improving pork quality.

## 1. Introduction

As global meat consumption continues to rise, consumers are increasingly concerned about the flavor and nutritional value of meat, making meat quality a critical factor influencing consumer choices. However, meat quality is closely linked to fat characteristics. Factors such as the distribution, type, and content of fat can significantly impact the overall flavor, texture, and nutritional value of meat [1]. Genetic, nutritional, and environmental factors primarily regulate fat traits, with genetic factors playing a pivotal role in fat deposition and distribution through key genes and molecular signaling pathways, including adiponectin, leptin, fatty acid synthase (*FASN*), the Wnt signaling pathway, and the mTOR signaling pathway [2,3,4,5,6]. These factors contribute to significant variations in intramuscular fat (IMF) among the different pig breeds, with IMF content correlating with breed characteristics. This variation may stem from differing transcript levels of the leptin receptor (*LEPR*) and fatty acid binding protein 3 (*FABP3*) genes [7]. Compared to commonly used lean pig breeds, Chinese local pig breeds exhibit notable advantages in fat deposition capacity, IMF content, and overall meat quality [8,9]. For instance, the IMF content in Tibetan and Laiwu (LW) pigs is significantly higher than that in Yorkshire pigs [10,11].

The Tibetan pig is a valuable local breed in China, predominantly found in Tibet, Qinghai, Gansu, and other high-altitude regions. The prolonged natural selection in harsh environments has resulted in enhanced disease resistance and fat deposition. Studies indicate that the IMF content in Tibetan pigs is considerably higher than that in Landrace and Yorkshire breeds [12]. Additionally, Tibetan pigs possess greater fat percentages and backfat thickness compared to lean pigs. Their subcutaneous fat also contains elevated levels of monounsaturated fatty acids, such as oleic acid (C18:1) and hexadecenoic acid (C16:1), which may enhance nutritional value due to benefits associated with cardiovascular health and lipid metabolism [13]. Differential fat depositions exhibit variations in morphology, as well as the cytokines and adipokines they express. In production, there is a desire for pigs to have lower subcutaneous fat while increasing intramuscular fat, which would lead to more efficient feed utilization and improved meat quality [14]. However, intramuscular fat and subcutaneous fat deposition are positively correlated, and some studies have utilized backfat thickness as a predictor of intramuscular fat content [15]. Consequently, addressing how to reduce subcutaneous fat deposition while increasing intramuscular fat content without compromising growth rates has become a key challenge in the pig industry. Given the varying economic values of different fat parts, researchers often pursue different breeding strategies. Although several genes, including *H-FABP*, *FTO*, and *PPARGC1A*, have been identified as regulators of intramuscular fat deposition in pigs, studies examining the differences in fat deposition across various body regions and their specific mechanisms remain limited [16,17,18]. Therefore, identifying functional genes that influence differential fat deposition and analyzing their regulatory mechanisms has emerged as a focal point in pork quality improvement research. Currently, RNA-seq technology is widely employed to identify candidate genes and biological pathways that regulate meat quality traits in livestock and poultry [19]. For example, Piorkowska et al. identified *FGL1* gene locus variants associated with fat deposition based on porcine liver RNA-seq data [20]. The integration of ATAC-seq and RNA-seq analyses has identified *PVALB*, *THRSP*, *HOXA9*, *EEPD1*, *HOXA10*, and *PDE4B* as potentially associated with fat deposition in pigs [21]. In this study, we investigate the phenotypes of the different fat deposits in Tibetan pigs at both macro and micro levels. Furthermore, we reveal significant differences in deposition between subcutaneous and intramuscular fat in Tibetan pigs through RNA-seq analysis. Additionally, we hypothesize a set of genes and signaling pathways that may influence differential fat deposition. This research not only provides critical insights into the unique fat deposition characteristics of Tibetan pigs but also contributes to a deeper understanding of the regulatory mechanisms involved in differential fat deposition.

## 2. Materials and Methods

### 2.1. Sample Collection

The experimental animals for this study were Tibetan pigs, a local breed in China. They were raised under controlled conditions on the experimental farm of the Institute of Animal Science at the Guangdong Academy of Agricultural Sciences for slaughter. They were kept indoors in pens, with an average area of 2–3 m^2^ per pig. The diet was provided twice daily and was manually timed. The experimental diets were formulated using corn and soybean meal as the primary ingredients. Crude protein levels, trace minerals, and vitamins were adjusted to align with the National Research Council (NRC 1998) guidelines for various growth stages. Environmental conditions, including temperature and humidity, were monitored to ensure consistency. Three 8-month-old female Tibetan pigs were randomly selected from the same feeding group, weighed, and slaughtered after a 24 h fasting period at an experimental animal slaughterhouse away from the farm. Following slaughter, samples of subcutaneous fat (abdominal fat and back fat) and intramuscular fat (left side at 3rd–4th ribs, longissimus dorsi) were collected, partially packed into freezing tubes, and immediately frozen in a liquid nitrogen tank for long-term storage at −80 °C. The remaining samples were preserved in 4% paraformaldehyde for tissue section preparation.

### 2.2. Adipose Tissue Section Preparation

Paraffin sections were prepared from harvested abdominal fat, back fat, and intramuscular adipose tissue. The preparation steps included: fixation, where the sections were placed in 4% paraformaldehyde (Thermo Fisher, Waltham, MA, USA) to preserve cell morphology; dehydration and transparency, where adipose tissue was dehydrated in a gradient using various ethanol (Yongda Chemical Reagent Co., Ltd., Tianjin, China) concentrations: 50% ethanol for 1 h, 70% ethanol for 1 h, 80% ethanol for 1 h, 90% ethanol for 1.5 h, and 95% ethanol for 1.5 h, followed by rendering the tissue transparent with xylene (Yongda Chemical Reagent Co., Ltd., Tianjin, China); wax dipping, where the transparent tissue blocks were embedded in melted paraffin wax (Beijing Solarbio Science & Technology Co., Ltd., Beijing, China); sectioning, where sections were cut with a paraffin slicer and placed in an oven at 60 °C for 12 h; deparaffinization, where sections were treated with xylene to remove paraffin; and rehydration, where sections were rehydrated in 95% ethanol for 5 min, 80% ethanol for 5 min, 70% ethanol for 5 min, and 50% ethanol for 5 min, followed by rinsing in ultrapure water for 5 min, repeated three times. Hematoxylin (Beijing Solarbio Science & Technology Co., Ltd., Beijing, China) and eosin (Beijing Solarbio Science & Technology Co., Ltd., Beijing, China) staining was performed, with hematoxylin staining the nuclei and eosin staining the cytoplasm. The stained sections were dehydrated again using an alcohol gradient and rendered transparent with xylene. Finally, the completed sections were imaged using a microscope (Nikon Y-TV55, Tokyo, Japan) at 100× and 400×.

### 2.3. RNA-Seq

To investigate transcriptomic differences between subcutaneous fat (abdominal fat and back fat) and intramuscular fat in Tibetan pigs, total RNA was extracted from the collected samples (0.1 g of each tissue) using the TRIzol method (Thermo Fisher, Waltham, MA, USA) and the sample size for each organization was set to three biological replicates. The purity and concentration of the total RNA were assessed using a NanoDrop Photometer N60 (Implen GmbH, Munich, Germany). The samples were then sent to Majorbio Bio-Pharm Technology Co. (Shanghai, China). for transcriptome sequencing. The sequencing experiment used the Illumina^®^ Stranded mRNA Prep, Ligation (San Diego, CA, USA) method for library construction. The sequencing library was sequenced on the NovaSeq X Plus platform (PE150) using the NovaSeq Reagent Kit (Illumina, San Diego, CA, USA). After quality control, the raw data were processed, and the reference genome (reference genome version: Sscrofa11.1; reference genome source: http://asia.ensembl.org/Sus_scrofa/Info/Index, accessed on 17 November 2022) was aligned using HiSat2 (http://ccb.jhu.edu/software/hisat2/index.shtml, accessed on 17 November 2022) software. Gene and transcript expression levels were quantified using RSEM (http://deweylab.github.io/RSEM/, accessed on 17 November 2022) software. Differential expression analysis was performed using the DESeq2 software (Version 1.24.0, http://bioconductor.org/packages/stats/bioc/DESeq2/, accessed on 17 November 2022). The default screening criteria for significantly differentially expressed genes are: FDR < 0.05 and |log2FC| ≥ 1. When a gene meets both conditions, it is considered a differentially expressed gene (DEG). In addition, functional-enrichment analysis including GO (GO, http://geneontology.org/, accessed on 17 November 2022) and KEGG (KEGG, https://www.kegg.jp/, accessed on 17 November 2022) were performed to identify which DEGs were significantly enriched in GO terms and metabolic pathways at Bonferroni-corrected *p*-value < 0.05 compared with the whole-transcriptome background. GO functional enrichment and KEGG pathway analysis were carried out by Goatools (https://github.com/tanghaibao/GOatools, accessed on 17 November 2022) and Python scipy (Version 1.0.0, https://scipy.org/install/, accessed on 17 November 2022) software, respectively.

### 2.4. RT-qPCR

Tissue samples from subcutaneous fat (abdominal fat and back fat) and intramuscular fat were extracted for total RNA using the TRIzol method, and RNA was reverse transcribed to cDNA according to the manufacturer’s instructions (TaKaRa, Tokyo, Japan). Quantitative PCR (qPCR) was performed using a Bio-Rad CFX96 (Bio-Rad, Hercules, CA, USA) with TB Green^®^ Premix Ex Taq™ II (Tli RNaseH Plus) (TaKaRa, Tokyo, Japan) to detect mRNA abundance. The qPCR reaction mixture consisted of 10 µL of 2× Taq Pro Universal SYBR qPCR Master Mix (Vazyme, Nanjing, China), 1 µL of cDNA template, 1 µL each of upstream and downstream primers, and 7 µL of water to make a total volume of 20 µL. The primers for *DDAH1*, *ADRA1B*, *SLCO3A1*, *THBS3*, and *GAPDH* were designed by NCBI and synthesized by Shanghai Sangon Biotech Co., with *GAPDH* serving as the housekeeping gene. The primer sequences are: *DDAH1*: 5′-CCGTGTTCTCGGGCACTTAC-3′ and 5′- GCCAAGATTTCAGCACCTCG-3′; *ADRA1B*: 5′-TGGTCATGTACTGCCGTGTC-3′ and 5′-TTGGCCTTCGTACTGCTGAG-3′; *SLCO3A1*: 5′-GCTGAGAGTGATCCCGAAGG-3′ and 5′-TGCAGGTGAACACAGGGTTT-3′; *THBS3*: 5′-AGCAAACGGAGCAGACCTAC-3′ and 5′-ACCGATGTCACTGCCTTGAG-3′; *GAPDH*: 5′-GAAGGTCGGAGTGAACGGATTT-3′ and 5′-TGGGTGGAATCATACTGGAACA-3′. The qPCR cycling conditions were as follows: initial denaturation at 95 °C for 30 s, followed by 40 cycles of denaturation at 95 °C for 5 s and annealing at 60 °C for 30 s. The relative expression of each gene was calculated using the formula 2^−ΔΔCT^.

### 2.5. Statistical Analysis

All data was statistically analyzed using SPSS 18.0. Data are expressed as mean ± standard error of the mean. Results were analyzed using one-way ANOVA or independent sample *t*-tests, with differences considered statistically significant at *p* < 0.05.

## 3. Results

### 3.1. Comparison of Histological Characteristics of Different Types of Adipose Tissue

**Figure 1:**
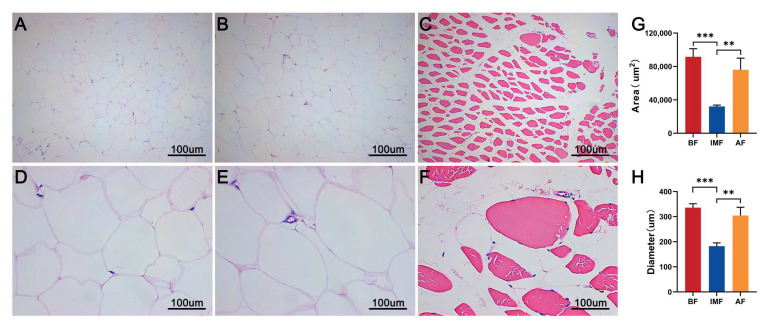
Morphological observation of paraffin sections: (**A**) abdominal fat at 100×; (**B**) back fat at 100×; (**C**) intramuscular fat at 100×; (**D**) abdominal fat at 400×; (**E**) back fat at 400×; (**F**) intramuscular fat at 400×; (**G**) statistics of adipocyte area; (**H**) statistics of adipocyte diameter; ** *p* < 0.01, *** *p* < 0.001.

To assess differences in fat deposition across various regions of the Tibetan pig, we first prepared hematoxylin and eosin (HE)-stained sections of subcutaneous adipose tissue (abdominal fat and back fat) and intramuscular adipose tissue. Microscopic examination revealed that adipocytes in the abdominal region were irregularly shaped, rounded, and densely packed, exhibiting a larger size (Figure 1A,D). The morphology of adipocytes in the back fat was similar to that of abdominal adipocytes (Figure 1B,E). In contrast, intramuscular adipocytes were smaller and heterogeneous in size, primarily located in the interstitial spaces of myocytes (Figure 1C,F). Additionally, we measured the area and diameter of these adipocytes and found that subcutaneous adipocytes were significantly larger than intramuscular adipocytes (Figure 1G,H).

### 3.2. Identification of Differentially Expressed Genes in Different Types of Adipose Tissue

**Figure 2:**
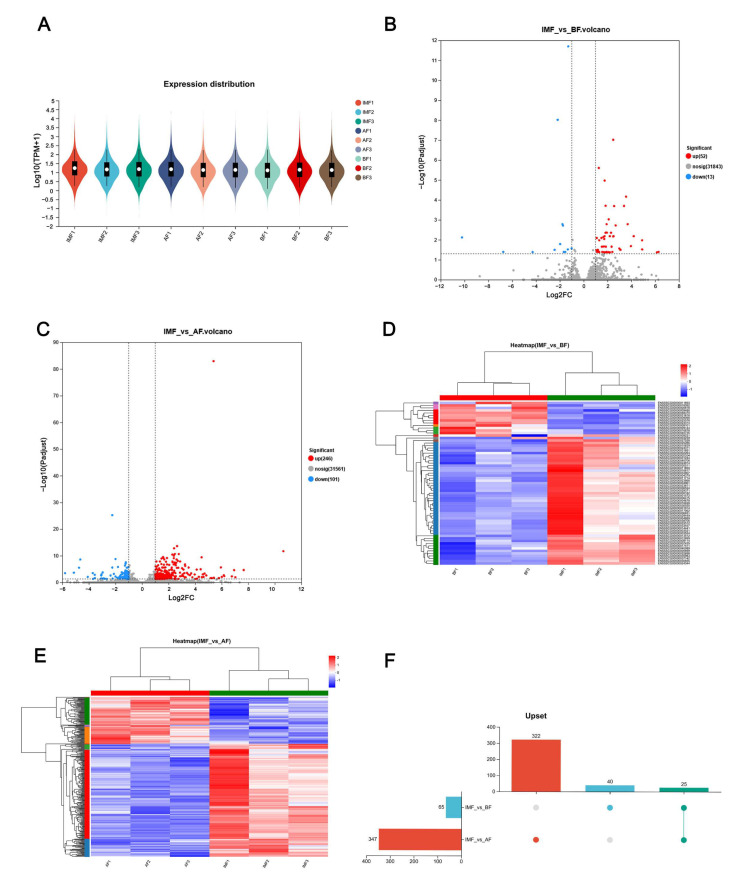
RNA-seq identifies the DEGs in Tibetan pigs’ subcutaneous fat and intramuscular fat. (**A**) Expression distribution plot between IMF, AF and BF; (**B**) IMF vs. BF volcano plot; (**C**) IMF vs. BF heat map; (**D**) IMF vs. AF volcano plot; (**E**) IMF vs. AF heat map; (**F**) IMF vs. BF and IMF vs. AF DEGs upset plot.

We utilized transcriptomics to identify genes that are differentially expressed in subcutaneous and intramuscular fat in Tibetan pigs, aiming to explore the mechanisms underlying the differences in fat deposition at various sites. Initially, we filtered the raw data from transcriptome sequencing to ensure accuracy. The results indicated a more homogeneous distribution of expression in abdominal fat, back fat, and intramuscular fat (Figure 2A), confirming the credibility of the sequencing data and ensuring the authenticity of subsequent results. Overall, RNA-seq analysis of Tibetan pig BF and IMF revealed a total of 65 DEGs, with 52 upregulated and 13 downregulated (Figure 2B). Additionally, 347 DEGs were identified by comparing AF and IMF, with 246 upregulated and 101 downregulated (Figure 2C). Subsequently, DEGs between the two groups were analyzed using cluster analysis, revealing the number of shared and independently owned DEGs. The UpSet plot indicated that 25 DEGs were shared between the two groups, with 40 DEGs unique to BF and IMF and 322 DEGs unique to AF and IMF (Figure 2F). Heatmap results demonstrated clear differences between BF and IMF, as well as between AF and IMF (Figure 2D,E).

### 3.3. Functional Annotation and Enrichment of DEGs

**Figure 3:**
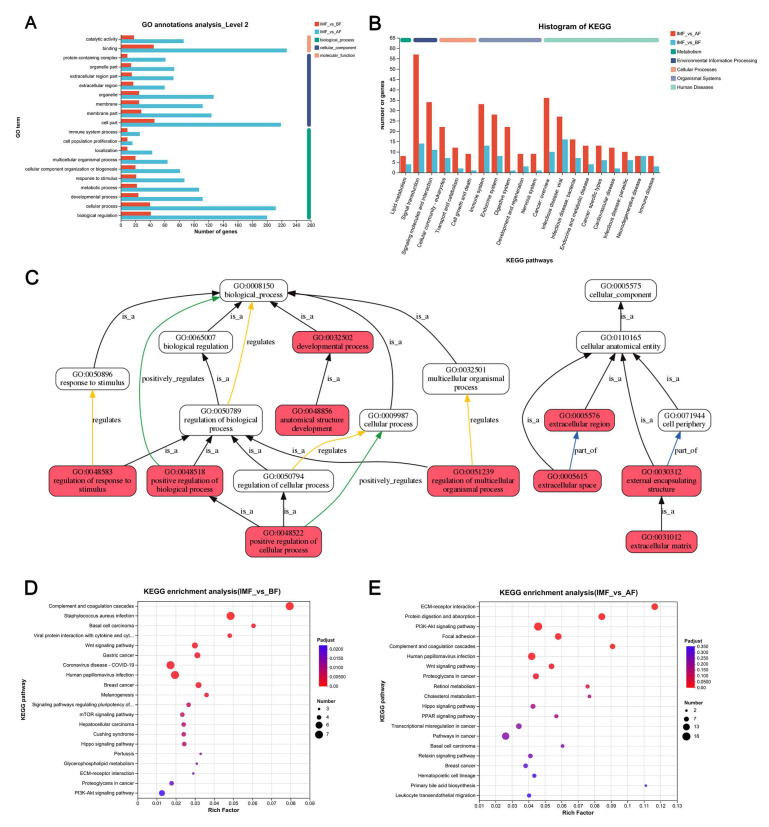
Enrichment analysis of DEGs. (**A**) GO annotation diagram of DEGs common to the two groups of BF vs. IMF and AF vs. IMF; (**B**) KEGG annotation diagram of DEGs common to the two groups of BF vs. IMF and AF vs. IMF; (**C**) DEGs GO schematic. Each box represents a GO term, and the red boxes represent significant enrichment. Black arrows indicate “is_a” relationships, i.e., subclass relationships; Yellow arrows indicate “regulates” relationships; Green arrows indicate “positively regulates” relationships. Blue arrows indicate “part of” relationships; (**D**) KEGG enrichment plot of BF vs. IMF group; (**E**) KEGG enrichment plot of AF vs. IMF group.

To enhance the comprehensiveness of the transcriptome data analysis, we employed Gene Ontology (GO) and Kyoto Encyclopedia of Genes and Genomes (KEGG) annotations to classify the functions of the differentially expressed genes in the two groups. GO annotation categorized the shared DEGs into three main categories: biological processes, cellular components, and molecular functions. Among these DEGs, biological processes represented the most abundant category, comprising 10 items. The top five enriched biological processes included biological regulation, cellular processes, developmental processes, metabolic processes, and responses to stimuli. The second most abundant category was cellular components, consisting of eight items, with the top five including cell parts, membrane parts, membranes, organelles, and extracellular regions. The molecular function category contained two items: binding and catalytic activity (Figure 3A). To visualize the functional roles of these DEGs, we constructed a GO schematic illustrating the pathways in which the differential genes were significantly enriched (Figure 3C).

KEGG annotation revealed that these DEGs were primarily associated with five major pathways: metabolism, environmental information processing, cellular processes, organismal systems, and human diseases (Figure 3B), reflecting their diverse biological roles. KEGG enrichment analysis indicated that the DEGs in the BF vs. IMF group were significantly enriched in the complement and coagulation cascades, the Wnt signaling pathway, signaling pathways regulating pluripotency of stem cells, the mTOR signaling pathway, glycerophospholipid metabolism, ECM–receptor interaction, and several other pathways (Figure 3D). The main enriched pathways of the DEGs between the AF vs. IMF groups included ECM–receptor interaction, protein digestion and absorption, the PI3K-Akt signaling pathway, proteoglycans in cancer, the Wnt signaling pathway, and others (Figure 3E).

Among the common 25 DEGs, three genes were upregulated (*DDAH1*, *ADRA1B*, and *ALDH1A2*), while 22 genes were downregulated (*CFB*, *PRDM8*, *CDS1*, *CADM3*, *WNT4*, *THBS3*, *PRSS12*, *CRIP1*, *FHL1*, *LGR5*, *SLCO3A1*, *TEDC1*, *MAMDC2*, *WNT10B*, *PLXDC1*, *TNXB*, *TPPP*, *PLTP*, *ITGA11*, *ADRA2A*, *SOBP*, and *pRG4*). These differentially expressed genes are primarily enriched in pathways related to human papillomavirus infection, breast cancer, gastric cancer, ECM–receptor interaction, the Wnt signaling pathway, the PI3K-Akt signaling pathway, and stem cell pluripotency regulatory signaling pathways.

### 3.4. Quantitative Fluorescent PCR to Verify Differentially Expressed Genes

**Figure 4:**
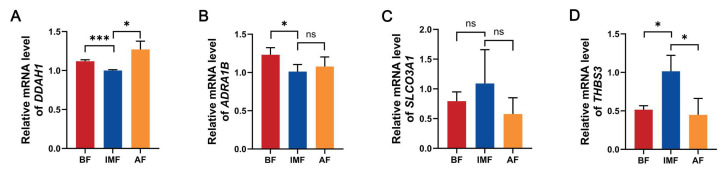
The mRNA expression levels of candidate genes in subcutaneous and intramuscular fat. (**A**) *DDAH1* mRNA expression level; (**B**) *ADRA1B* mRNA expression level; (**C**) *SLCO3A1* mRNA expression level; (**D**) *THBS3* mRNA expression level. With GAPDH as the housekeeping gene, * *p* < 0.05, *** *p* < 0.001, ns means the difference is not significant.

To further validate the accuracy of the RNA-seq results, we selected four genes (*DDAH1*, *ADRA1B*, *SLCO3A1*, *THBS3*) among the 25 shared DEGs that may be related to growth, development, and fat metabolism for qPCR analysis. We found that the mRNA expression of *DDAH1* was generally higher in subcutaneous adipose tissue (BF and AF) than in intramuscular adipose tissue (Figure 4A). In contrast, only BF showed a significant difference from IMF in *ADRA1B* expression (Figure 4B). Although *SLCO3A1* did not exhibit a significant difference between the two adipose tissues, it displayed an overall decreasing trend in subcutaneous adipose tissue (Figure 4C). Conversely, *THBS3* expression was generally higher in IMF than in subcutaneous adipose tissue (Figure 4D). These results are consistent with the RNA-seq data, indicating that the RNA-seq findings are reliable.

## 4. Discussion

Fat deposition is a key factor affecting pork yield, grading and overall meat quality. Tibetan pigs have superior fat deposition capacity due to their unique genetic characteristics. Currently, numerous studies have focused on the mechanisms underlying differences in fat deposition traits between Tibetan pigs and other pig breeds [8,22]. However, research on the growth patterns of specific fat deposition sites in Tibetan pigs remains limited. Therefore, our study highlights the scientific significance of investigating the mechanisms of fat metabolism in Tibetan pigs. In this study, we observed significant morphological differences between subcutaneous and intramuscular adipocytes in Tibetan pigs. The area and diameter of subcutaneous adipocytes (abdominal fat and back fat) were significantly larger than those of intramuscular adipocytes. This finding aligns with previous reports on fat deposition in the Huai pig [23]. The notable difference between subcutaneous and intramuscular adipocytes may arise from the primary role of subcutaneous fat as a major energy storage site [24]. Additionally, subcutaneous fat serves as the primary storage location for free fatty acids and triglycerides in the body. Only when its storage capacity is saturated are excess free fatty acids and triglycerides redirected to other sites, forming intramuscular fat, such as that found in the longissimus muscle [25,26,27]. Therefore, we speculate that the distinctive fat distribution in Tibetan pigs significantly contributes to their superior meat quality and unique flavor.

With the advancements in high-throughput sequencing technology, transcriptomics has become an essential tool for analyzing complex genetic traits in agricultural animals [5]. In this study, we performed RNA-seq to analyze the transcriptomes of subcutaneous and intramuscular adipose tissues in Tibetan pigs, identifying a substantial number of expressed transcripts. To gain deeper insights into the characteristics of these adipose tissues, we compared transcript expression levels between the two tissue types. Our results revealed 65 DEGs in the comparison of BF versus IMF, with 52 upregulated and 13 downregulated genes. In contrast, 347 DEGs were identified in the comparison of AF versus IMF, including 246 upregulated and 101 downregulated genes. In total, 25 DEGs were co-expressed in both subcutaneous and intramuscular fat. KEGG enrichment analysis indicated that the shared DEGs between the two groups were primarily enriched in lipid metabolism-related pathways, including the Wnt signaling pathway, mTOR signaling pathway, glycerophospholipid metabolism, ECM–receptor interaction, and PI3K-Akt signaling. The Wnt signaling pathway plays a fundamental role in the development and function of adipose tissue, affecting adipocyte differentiation and maturation while also regulating inflammatory responses and energy homeostasis in adipose tissue [5]. Additionally, several pathways associated with metabolic diseases, such as amino acid and fatty acid metabolism, insulin signaling pathways, and obesity-related pathways, may also influence subcutaneous and intramuscular fat deposition and metabolism [28]. Finally, we conducted further analyses by selecting representative genes from these pathways. Among the four tested genes, we observed that *DDAH1* mRNA expression was significantly higher in subcutaneous adipose tissue (BF and AF) than in intramuscular adipose tissue.

Research indicates that *DDAH* consists of two subunits, *DDAH1* and *DDAH2*, which are associated with cardiovascular diseases induced by hypercholesterolemia, hypertension, abdominal obesity, and diabetes mellitus [29,30]. Furthermore, *DDAH1* enhances nitric oxide synthase (NOS) activity by degrading asymmetric dimethylarginine (ADMA), leading to increased nitric oxide (NO) production. NO influences the differentiation and fatty acid metabolism of adipose precursor cells by modulating signaling pathways and transcription factors involved in adipogenesis [31,32]. During adipogenesis, appropriate levels of NO can regulate the activity of certain adipose-related transcription factors, thereby promoting or inhibiting adipogenesis [33]. Moreover, in vivo studies have demonstrated that overexpression of *DDAH1* mitigates metabolic dysfunctions, such as lipid accumulation and inflammation, in high-fat-fed mice and mice with non-alcoholic fatty liver disease (NAFLD) [34].

The *ADRA1* family includes *ADRA1A* and *ADRA1D* in addition to *ADRA1B*. These receptors regulate various physiological processes by binding to catecholamines such as epinephrine and norepinephrine, activating intracellular signaling pathways. Among these, *ADRA1A* is the predominant α1-adrenergic receptor subtype in the adipose tissue vasculature of obese individuals and has been implicated in regulating vascular tone and obesity-driven hypertension [35]. Obesity is also associated with the proliferation and differentiation of adipose precursor cells. In ovarian granulosa cells of rats with polycystic ovary syndrome (PCOS), *SLCO3A1* mRNA expression was significantly higher than in normal cells, suggesting its potential role in lipid metabolism and obesity-related conditions [36]. Furthermore, studies have reported that *SLCO3A1* is associated with lipid metabolism-related genes and immune cell infiltration in single-cell transcriptomic analyses [37].

*THBS3* is an adhesion glycoprotein that functions in several biological processes, primarily mediating cell–extracellular matrix interactions [38]. *THBS3* has been identified as a candidate gene influencing drip loss in pork, as revealed by combined transcriptomic and proteomic analyses. Since drip loss correlates closely with IMF content, *THBS3* may also play a role in fat deposition [39]. In our study, *THBS3* expression was highest in IMF tissues. Additionally, our sequencing results indicated that *THBS3* was primarily enriched in pathways related to extracellular matrix (ECM)–receptor interaction and PI3K-Akt signaling. Reports suggest that the ECM–receptor interaction pathway impacts meat quality by regulating the metabolic processes of intramuscular fat cells [40]. The PI3K-Akt signaling pathway plays a key role in regulating the lipid metabolism, affecting multiple genes related to the lipid metabolism, various lipid metabolism organs, and the coordinated functioning of multiple metabolic activities [41]. These results highlight the potential role of *THBS3* in adipose tissue development and lipid metabolism.

Notably, some DEGs were found to be enriched in immune-system signaling pathways in our study. Signaling pathways of the immune system have been reported to interact with fat deposition and lipid metabolism in a complex manner, possibly through chronic low-grade inflammatory states, immune cytokine secretion, and other pathways. For instance, a chronic low-grade inflammatory state typically occurs with obesity, characterized by an increased proportion of M1-type (pro-inflammatory) macrophages and a decreased proportion of M2-type (anti-inflammatory) macrophages in adipose tissue. This change significantly increases the secretion of pro-inflammatory factors (e.g., TNF-α and IL-6), which further affect fat accumulation, lipid metabolism, and systemic insulin sensitivity [42]. Furthermore, the TLR signaling pathway, particularly TLR4, serves as a bridge between the immune system and adipocytes in regulating the lipid metabolism. A diet high in saturated fatty acids significantly activates the TLR4 signaling pathway, inducing the release of pro-inflammatory factors from adipocytes and circulating immune cells, thereby influencing fat metabolism and insulin sensitivity [43]. Future studies may provide further insights into these mechanisms.

## 5. Conclusions

In summary, our study identified significant morphological differences in fat cells between subcutaneous and intramuscular fat in Tibetan pigs. These differences may reflect the distinct characteristics of these two types of fat regarding lipid storage and metabolic functions. Additionally, transcriptome analysis revealed differentially expressed genes and their enrichment in lipid metabolic pathways, including the Wnt and PI3K-Akt signaling pathways. We further identified candidate genes that may influence fat deposition, such as *DDAH1*, *ADRA1A*, *SLCO3A1*, and *THBS3*. These findings provide valuable insights into the mechanisms of adipogenesis and lipid metabolism. However, further research is necessary to elucidate the specific roles of these genes in adipose tissue differentiation, lipid storage, and metabolic regulation.

## Data Availability

The original contributions presented in this study are included in the article. Further inquiries can be directed to the corresponding authors.

## Abbreviations

The following abbreviations are used in this manuscript:

IMF	Intramuscular fat
DEG	Differentially expressed gene
qPCR	Quantitative PCR
HE	Hematoxylin and eosin
GO	Gene Ontology
KEGG	Kyoto Encyclopedia of Genes and Genomes
AF	Abdominal fat
BF	Back fat
